# Spatial anxiety mediates the sex difference in adult mental rotation test performance

**DOI:** 10.1186/s41235-020-00231-8

**Published:** 2020-07-25

**Authors:** Daniela Alvarez-Vargas, Carla Abad, Shannon M. Pruden

**Affiliations:** 1 School of Education, University of California Irvine, Irvine, CA USA; 2 Psychology Department, Florida International University, Miami, FL USA

**Keywords:** Sex differences, Mental rotation, Spatial anxiety, Trait anxiety, Mediation, Exploratory factor analysis

## Abstract

Mental rotation ability is associated with successful advances in STEM (science, technology, engineering, and mathematics) education and occupations. Meta-analyses have shown consistent sex disparities in mental rotation, where men outperform women on one measure of mental rotation ability, the *Mental Rotations Test* (*MRT*). Spatial anxiety, or the fear and apprehension felt when completing a task that requires spatial thinking, was proposed as a mechanism explaining the relation between sex and mental rotation test performance. This study modified the *Spatial Anxiety Scale* (*SAS*) to include questions about how anxious individuals feel when they must mentally rotate items to accomplish a task (e.g., playing Tetris). An exploratory factor analysis was conducted to assess the factorial structure of the modified spatial anxiety scale. Three factor loadings were extracted representing the ability to navigate, mentally rotate objects, and visualize objects. Furthermore, we analyzed the role of spatial anxiety and trait anxiety as potential mediators of the relation between participant sex and mental rotation performance. Spatial anxiety partially mediated the link between the sex of the participants and the MRT performance controlling for trait anxiety. Only navigation and mental rotation anxiety significantly mediated the relation between participant sex and mental rotation performance. We posit spatial anxiety as a barrier to efficient and accurate spatial thinking, and suggest that reducing spatial anxiety has the potential to improve spatial skills and reduce sex differences in mental rotation test performance. To ascertain this, an experimental design can determine whether a reduction in spatial anxiety causes changes in mental rotation test scores.

## Significance statement

Spatial thinking may be the gatekeeper to success in STEM entry-level courses (Uttal & Cohen, 2012; Wai, Lubinski, & Benbow, 2009). Several meta-analyses have demonstrated that mental rotation test scores demonstrate the largest sex differences (Uttal et al., 2013; Voyer, Voyer, & Bryden, 1995). Many researchers have explored the mechanisms through which sex differences may influence differential selection into the STEM fields to inform interventions that can reduce sex disparities (Estes & Felker, 2012; Levine, Vasilyeva, Lourenco, Newcombe, & Huttenlocher, 2005; Nazareth, Herrera, & Pruden, 2013; Neuburger, Ruthsatz, Jansen, & Quaiser-Pohl, 2015; Reilly & Neumann, 2013; Thoresen et al., 2016). We contributed to this work by highlighting spatial anxiety as a mechanism that may be contributing to sex differences in mental rotation ability. Similar to Lyons et al. (2018), we found evidence of a three-factor structure (i.e., navigation, mental rotation, visualization) of spatial anxiety despite (1) using different theoretical approaches and (2) using different scale items that measured spatial anxiety that laypeople may experience in their daily lives. Importantly, the current study is novel in its examination of sex differences in mental rotation and whether spatial anxiety potentially mediates the relation. Furthermore, we explored whether each of the three specific sub-factors of spatial anxiety mediated sex differences in mental rotation. Our results offer the promise of identifying specific types of spatial anxiety as targets for future experimental or intervention research to potentially reduce sex differences in mental rotation.

## Background

Spatial thinking consists of the conceptualization and manipulation of information about 2D and 3D objects in the world such as deriving information about shapes, object-to-object relations, frames of reference, and location (Newcombe & Shipley, 2015). Numerous daily activities, such as packing a suitcase, rearranging furniture, finding your car in a parking garage, and drawing a graph, require spatial thinking. Importantly, longitudinal studies show that individuals who pursue and achieve success in Science, Technology, Engineering, and Mathematics (STEM) fields on average have higher scores on measures of spatial ability (Hegarty, Crookes, Dara-Abrams, & Shipley, 2010; Humphreys, Lubinski, & Yao, 1993; Shea, Lubinski, & Benbow, 2001; Wai et al., 2009). Spatial tests are used to predict performance (Lohman, 1996) in professions including engineering, physical sciences, geosciences, and geography. Furthermore, professionals in these fields have higher self-ratings of their spatial skills, including mental rotation and navigation skill, compared to those in other professions (Hegarty et al., 2010). Overall, spatial ability is strongly associated with entry into and success in the STEM fields (Humphreys et al., 1993; Shea et al., 2001; Wai et al., 2009) and thus is a promising skill to encourage through education.

### Mental rotation: how mentally rotating objects is important to success in school

Spatial skills are required in daily school activities ranging from early childhood with the introduction of shapes to increasingly complex forms of problem solving found higher education courses. One particular spatial skill, mental rotation, has received a great deal of attention as it not only shows links to later math and science abilities but also consistently illustrates sex differences in adulthood, with males outperforming females (Voyer et al., 1995). Mental rotation is the ability to mentally rotate 2D and 3D shapes and objects. Mental rotation has been shown to be necessary to understanding numerical magnitudes (Casey, Dearing, Vasilyeva, Ganley, & Tine, 2011; Gunderson, Ramirez, Beilock, & Levine, 2012), applying measurement formulas, completing basic calculations (Casey et al., 2011), and interpreting diagrams of chemical reactions (Pribyl & Bodner, 1987). Furthermore, mental rotation is correlated with performance in geometry and verbal math problems (Delgado & Prieto, 2004), as well as with general math aptitude, as measured by the *Scholastic Aptitude Test for Math* (*SAT–M*), a college entrance exam used to predict success in math and science college courses (Casey, Nuttal, Pezaris & Benbow, 1995). Finally, Cheng and Mix (2014) demonstrated that children who received training in mental rotation improved in their ability to solve missing term problems (e.g., 6 + _ = 10), suggesting that interventions targeting mental rotation ability may show transfer effects to math. Together, these findings suggest the critical importance of spatial skills, specifically mental rotation, for success in math and academic performance.

### Sex differences in mental rotation

One of the most reliable sex differences found in cognitive psychology is the one found in mental rotation ability. Markedly, males consistently outperform females on the *Mental Rotations Test* (*MRT*), which was originally developed by Vandenberg and Kuse (1978) and later redrawn by Peters et al. (1995). In a typical mental rotation task, participants are shown a target figure constructed of 2D or 3D cubes and some stimulus figures that are correctly rotated versions of the target figure and some stimulus figures that are mirror images of the target figure. Participants must then choose which of the stimulus figures are matches to the target figure. By adulthood, sex differences are well-established across the literature (Maccoby & Jacklin, 1974; Uttal et al., 2013) and are found to vary in magnitude with males having an even greater advantage over females when the test is timed (Voyer, 2011) or when it utilizes 3D versus 2D figures (Voyer et al., 1995). The ontogeny of sex differences in mental rotation tasks is still unknown. Some have found a male advantage in children as young as 3 months of age (Moore & Johnson, 2008, 2011; Quinn & Liben, 2008, 2014). Others have identified emerging sex differences during preschool (Levine, Huttenlocher, Taylor, & Langrock, 1999; Levine, Ratliff, Huttenlocher, & Cannon, 2012). Abad, Odean, and Pruden (2018) found emerging sex differences in mental rotation ability gains made during the prekindergarten year, further highlighting the importance of examining the development of sex differences over time. However, several studies have found no sex differences in preschool and school-aged children (Caldwell & Hall, 1970; Estes, 1998; Frick, Ferrara, & Newcombe, 2013; Lachance & Mazzocco, 2006; Lehmann, Quaiser-Pohl, & Jansen, 2014; Verdine, Golinkoff, Hirsh-Pasek, & Newcombe, 2017). Evidence from a meta-analysis of cognitive sex differences in spatial abilities demonstrated that sex differences in mental rotation stabilize after puberty (Voyer et al., 1995).

As a field, we have identified numerous mechanisms potentially explaining the reported male advantage in mental rotation ability in adulthood. For example, evidence exists that preference for sports and toys with a spatial component (e.g., Legos, blocks) relates to sex differences on the Mental Rotations Test (Voyer, Nolan, & Voyer, 2000). Other studies have shown that experiences with spatial activities (e.g., playing with a Lego blocks; Nazareth et al., 2013), beliefs in gender-roles/gender-stereotypes (Neuburger et al., 2015; Reilly & Neumann, 2013), activation of the negative stereotype that men are better than women at mental rotation (Levine et al., 2005), confidence about performance (Estes & Felker, 2012), and even general anxiety felt while taking the test (Thoresen et al., 2016), all explain sex differences in mental rotation performance. Recently, calls have been made for more research addressing whether certain affective factors, such as domain-specific anxiety (i.e., spatial anxiety), explain some of these sex differences (e.g., Lauer, Esposito, & Bauer, 2018; Levine, Foley, Lourenco, Ehrlich, & Ratliff, 2016; Pruden et al., 2020).

### On the malleability of mental rotation ability

Spatial skills are malleable, with marked improvement showing in mental rotation ability after training (Casey et al., 2008; Hsi, Linn, & Bell, 1997; Sorby, Casey, Veurink, & Dulaney, 2013; Tzuriel & Egozi, 2010; Uttal et al., 2013). A meta-analysis of 217 studies conducted by Uttal et al. (2013) showed that spatial skills respond to training, that training effects are durable, and that you can see transfer effects from training in one spatial skill to another spatial skill. Critically, results also showed that, although males have an advantage in mental rotation ability, both males and females improve equally during training. Spatial training has also improved the ability to rotate 2D (Tzuriel & Egozi, 2010) and 3D shapes (Hsi et al., 1997), and in turn reduce reported sex differences in mental rotation performance. Sorby et al. (2013) implemented a spatial training curriculum for undergraduates who showed improvement in both their mental rotation ability and transfer effects to their calculus grades. STEM course grades also improved following a similar intervention by Miller and Halpern (2013); however, the effects diminished after 6 months. Presently, we have not established which form of spatial training would be the most effective in reducing individual and sex differences on mental rotation performance. Part of the problem in identifying effective intervention designs is determining which mechanisms or factors cause individual and sex differences.

Mechanisms that may explain individual differences in mental rotation have been proposed as promising avenues to bridge sex differences. One such mechanism can include training individuals to be more flexible in their strategy-use when performing spatial problem solving. Flexible strategy-use, such as forming a mental image or decomposing the image to determine how it would look in another rotation, is related to success on mental rotation tasks (Nazareth, Killick, Dick, & Pruden, 2019; Stieff, Dixon, Ryu, Kumi, & Hegarty, 2014). These findings on training spatial skills suggest that it is worthwhile to identify additional factors that explain sex differences in mental rotation ability to develop interventions that reduce sex differences in performance. In response, the current study has been designed to explore the negative influence of spatial anxiety on sex differences in mental rotation performance as a potential mechanism for future targeted interventions.

### Spatial anxiety as a potential mediator of sex differences in mental rotation ability

One particular factor worth exploring as a mechanism that may explain sex differences in mental rotation ability is anxiety. Broadly, anxiety is marked by negative thoughts like worrying or emotions of apprehension and fear (Spielberger, 2010). Spatial anxiety, a multi-faceted, domain-specific type of anxiety, is defined as anxiety about performing spatial tasks (e.g., navigation, wayfinding, mentally manipulating or rotating objects, perspective taking; Lawton, 1994; Ramirez, Gunderson, Levine, & Beilock, 2013). Domain-specific anxiety, like spatial anxiety, may be a potential mediator of sex differences seen in mental rotation ability. Research suggests that both test anxiety and generalized/trait anxiety impact a number of academic skills, including spatial ability. A review of 126 studies demonstrated that individual differences in test anxiety negatively correlated to academic performance (Seipp, 1991). The analyses provided that the most accurate measurement of anxiety must be specific to the domain of interest if it is to be predictive of academic performance. Thus, anxiety for spatial tasks should be most related to performance on spatial tasks.

We consider the impact of anxiety on working memory while viewing working memory as a multiple-component system, as proposed by Baddeley and Hitch (2000). Because anxiety induces thoughts and ruminations that are believed to require the use of verbal working memory, anxiety can interfere with performance on tasks that require the use of verbal working memory resources (Beilock & Carr, 2005; Kane & Engle, 2002). Spatial reasoning places a heavy demand on working memory (Kyllonen & Christal, 1990). Specifically, evidence exists that anxiety and spatial reasoning require the use of the visuo-spatial component of working memory capacity (Gabriel, Hong, Chandra, Lonborg, & Barkley, 2011; Hyun & Luck, 2007). Because a link exists between trait anxiety and working memory, and spatial reasoning tasks place a high demand on verbal and visuo-spatial working memory resources, anxiety plausibly could lower performance on the mental rotations test due to reduced processing efficiency, as processing efficiency theory (Eysenck & Calvo, 1992) would predict. Moreover, reduced processing efficiency can contributed to lower performance on a task like the Mental Rotations Test because it is a timed test (Vandenberg & Kuse, 1978). Although the use of more optimal strategies could aid the reduction of processing effectiveness during mental rotation tasks, female participants demonstrate less strategy flexibility during mental rotation tasks than male participants (Nazareth et al., 2019). Thus, processing efficiency should be negatively impacted by high working memory demands, and processing effectiveness should be negatively impacted by lower strategy flexibility, thereby explaining the negative relation between anxiety and performance.

Work by Gunderson et al. (2012) finds that spatial anxiety in children is negatively related to their performance on a mental rotation task but only for those children with strong working memory skills. This spatial anxiety by working memory interaction was only evident in girls, not boys, suggesting that girls with the highest working memory may be at the greatest disadvantage. Evidence also exists that teacher spatial anxiety is related to student spatial ability. Gunderson, Ramirez, Beilock, and Levine (2013) also finds that higher levels of spatial anxiety in first and second grade teachers in the beginning of the school year is correlated with student spatial skills at the end of the school year. Together, these findings point to spatial anxiety as having a potential role in explaining individual differences in mental rotation performance.

### Measuring spatial anxiety: the Spatial Anxiety Scale (SAS)

The most widely used measure of spatial anxiety for adults is the eight-item *Spatial Anxiety Scale (SAS*), created by Lawton in 1994, to address whether gender differences in adult way-finding strategies were related to anxiety about navigation or way-finding in the environment. When asked to rate their anxiety about various navigation/way-finding situations, female participants reported higher spatial anxiety than their male counterparts (Lawton, 1994; also see Vieites, Pruden, & Reeb-Sutherland, 2020). Spatial anxiety was not only significantly related to adult orientation strategy use on a self-report wayfinding strategy scale (Lawton, 1994; also see Vieites et al., 2020) but also to adult performance on a mental rotation task (i.e., the Vandenberg and Kuse (1978) Mental Rotations Test) and a spatial perception task (i.e., a Piagetian water-level task). Thus, these findings suggest a role for way-finding anxiety on way-finding strategy and perhaps even small-scale spatial abilities like mental rotation.

More recent work examining spatial anxiety has expanded and adapted the SAS to include questions pertaining to mental rotation and visualization of objects. Malanchini et al. (2017) conducted a study with over 1400 college-aged twins to explore the factor structure of spatial anxiety. A 10-item spatial questionnaire, of which 7-items were loosely based on the SAS, was developed and included questions on navigation/way-finding (e.g., “trying a new shortcut without using a map,”) and rotation/visualization (e.g., “having to rotate objects in your mind” and “having to complete a complex jigsaw puzzle”). Analysis showed that spatial anxiety is a multifactorial construct consisting of two separate factors: one factor for navigation/way-finding anxiety and another factor for rotation/visualization anxiety. Malanchini and colleagues argue that these results highlight the need to include spatial anxiety items addressing rotation/visualization anxiety in future studies.

Rather than building from the SAS questionnaire, Lyons et al. (2018) developed a new theoretically-motivated spatial anxiety questionnaire for adults using an iterative survey design. Initially, 130 questions that addressed four categories of spatial ability, including intrinsic-static (i.e., object imagery), intrinsic-dynamic (i.e., mental rotation/manipulation), extrinsic-static (i.e., map reading or spatial scaling), and extrinsic-dynamic (i.e., navigation/way-finding) were developed and tested on 64 adult participants. After items that produced no variability had been removed and an exploratory factor analysis had been conducted, three factors—navigation, mental rotation/manipulation, and object imagery (eight items addressing each factor or a total of 24 items)—were identified. With this newly developed 24-item spatial anxiety questionnaire, Lyons and colleagues recruited 251 college students and tested their navigation, mental rotation/manipulation, and object imagery skills relative to each type of spatial anxiety (2018). The results showed that females had higher navigation and mental rotation/manipulation anxiety, and that each factor of anxiety (navigation, mental rotation/manipulation, object imagery) was significantly related to performance on the respective types of spatial skills, even when a control was used for general (trait) anxiety. What remains unclear, however, is whether sex differences in mental rotation are explained or mediated by different types of spatial anxiety. We address this question in the present study by examining spatial anxiety (as both a single construct and as a construct that consists of two distinct factors: mental rotation and navigation) and its relation to sex differences in adult mental rotation ability.

### Current study

To gain a better understanding of the established link between spatial anxiety and mental rotation performance (Lawton, 1994; Lyons et al., 2018), we explore whether sex differences seen in adult mental rotation performance can be explained by spatial anxiety. To do so we used the SAS (Lawton, 1994), the factor that may best explain individual differences in mental rotation ability, while retaining the original questions about navigation and including additional questions specifically about mental rotation. Importantly, we were careful to include items that the layperson might encounter during a typical, everyday experience, rather than items experienced only in the STEM disciplines (Lyons et al., 2018). We administered an online survey that included the Mental Rotations Test (MRT; Peters et al., 1995); a modified version of the Spatial Anxiety Scale (M-SAS; original SAS by Lawton, 1994) which included new items about mental rotation anxiety; and the *State-Trait Anxiety Inventory Trait* subscale *(STAI-Trait*; Spielberger, Gorsuch, Lushene, Vagg, & Jacobs, 1983) as a measure of general anxiety. Like Malanchini et al. (2017), we first examined the factor loading structure of our *Modified Spatial Anxiety Scale (M-SAS)* to explore whether specific items load onto factors that have to do with navigation and mental rotation. Next, we explored whether spatial anxiety is one comprehensive construct that includes all items kept after the exploratory factor analysis on the M-SAS, thereby mediating sex differences in mental rotation performance. Finally, using our factor loadings, we investigated whether those items specifically pertaining to mental rotation mediate sex differences in mental rotation performance.

Thus, the aims of the current study were three-fold: (1) to create a Modified Spatial Anxiety Scale (M-SAS) with items that correspond to both navigation and mental rotation and explore the factor structure of the M-SAS, (2) to examine whether sex differences in MRT performance are mediated by individual differences in spatial anxiety, and (3) to distinguish whether spatial anxiety, as a comprehensive construct or a specific subfactor of spatial anxiety (i.e., mental rotation or navigation), mediates the sex difference in performance on the MRT.

We predicted, based on the previous findings by Lyons et al., 2018 and Malanchini et al., 2017, that our M-SAS will consist of at least two separate factors, each one representing a type of navigation anxiety and mental rotation anxiety. We also anticipated that a comprehensive or global measure of spatial anxiety from the M-SAS that collapses across all items kept after the exploratory factor analysis will mediate the sex difference in MRT scores, even after controlling for general (trait) anxiety. Finally, we hypothesized that those items corresponding to mental rotation anxiety factor will mediate sex differences in MRT performance, but the other navigation anxiety factor and trait anxiety will not mediate sex differences in MRT performance.

## Method

### Participants

Complete data were gathered from 517 of 659 undergraduate student online survey participants (357 females and 160 males between the ages of 18 to 33 years (*M* = 21.01, *SD* = 2.56) recruited from a large research university in South Florida. A survey created through Qualtrics–– a survey creation and data management platform–– was posted on the university’s psychology research participation system from October to December 2016. Students volunteered to take the survey for one extra credit point that could then be applied to any participating psychology course. The courses allowing extra credit were not manipulated by the authors of this study and ranged from *Introduction to Psychology* to upper-division specialty courses. On average, participants took 23.27 min (*SD* = 11.54) to complete the full survey. Participants represented a racially diverse population with 68% Hispanic/Latino/Spanish origin, 10.2% Black/African American, 9.3% White/Caucasian, 3% Asian, 8.2% mixed-race, and 1.6% other.

From a total of 659 participants, 132 were excluded for survey incompletion (*n* = 35) and failure to respond to attention checks correctly (*n* = 97). A criterion of α = .001 with 11 variables with a critical χ^2^ = 31.264 was used to assess the ten most outlying cases within each group. As a result, ten multivariate outliers were identified and removed.

### Measures

The survey included a demographic questionnaire with questions regarding the number of years spent in higher education, participant sex, participant age, the number of math or statistics courses taken in college, and current cumulative grade point average. The demographic variables were entered as covariates for all the analyses with the exception of participant sex, which was treated as an independent variable. After they had completed the demographic questionnaire, participants were given the following measures, in a fixed order, to complete at their own pace: the Mental Rotations Test (MRT; Peters et al., 1995), the Modified Spatial Anxiety Scale (M-SAS; original SAS by Lawton, 1994), and the State-Trait Anxiety Inventory (STAI-Trait; Spielberger et al., 1983). The MRT has traditionally been administered under a time constraint, which is a characteristic of the test that has yielded the largest sex differences in performance (Voyer, 2011). However, to ensure that sex differences found in the current study were not confounded by the time constraints, the participants were allowed to take as much time as needed.

#### Mental Rotations Test (MRT)

The *Revised Vandenberg and Kuse Mental Rotations Test*, *Form A* (*MRT*; Peters et al., 1995) was used to measure participants’ mental rotation ability. Each of the 24 items (four practice trials; 20 test trials) consisted of five 3D cubed figures. The left-most figure is the target figure. The four figures on the right are test figures comprised of vertically rotated configurations of the target figure. Of the four adjacent test figures, only two are matches to the target figure, as they are simply rotated along the vertical and/or horizontal axis. The other two test figures are non-matches to the target figure, as they are rotated mirror images of the target figure. Participants were instructed to identify the two test figures that are matches to the target figure. Participants indicated their responses through mouse clicks on the images of the figures on the computer screen.

During the MRT, participants were first shown four practice trials in which they were asked to select the two figures that matched the target figure and were provided feedback about the accuracy of their responses. After the four training trials, participants completed the full 20-item test trials, for which they could receive one point per trial only if they had selected the two correct matches in that trial. If a participant chose one correct match and one incorrect alternative, they were given 0 points for that trial. This resulted in an MRT (mental rotation) score ranging from 0 to 20.

#### Modified Spatial Anxiety Scale (M-SAS)

The Modified Spatial Anxiety Scale (M-SAS) was used to measure the anxiety students feel in situations that require the use of mental rotation and navigation. The M-SAS included items regarding situations that would require small-scale mental rotation (such as building a tent), in addition to Lawton’s original items regarding situations that require navigation skills, such as driving in an unfamiliar neighborhood to find a house (Lawton, 1994). New M-SAS items were generated by the authors and are presented in Table 1. The final M-SAS contained 21 questions. Participants were asked to report how much being in each situation bothered them on a 4-point scale where 1 = Not at All, 2 = Mildly, 3 = Moderately, and 4 = Severely. Participants indicated their response with a mouse click on the response boxes to the right of each item. This resulted in an M-SAS (spatial anxiety) score ranging from 21 to 84. We also calculated scores for mental rotation anxiety, navigation anxiety, and visualization anxiety to examine these constructs separately; we use our exploratory factor analysis as evidence to inform which items to include in the mental rotation anxiety factors, navigation anxiety factor, and visualization anxiety factor.

**Table 1:** Initial Modified Spatial Anxiety Scale (M-SAS) items

1. Leaving a store that you have been to for the first time and deciding which way to turn to get to a destination^a^	
2. Finding your way out of a complex arrangement of offices that you have visited for the first time^a^	
3. Pointing in the direction of a place outside that someone wants to get to and has asked you for directions, when you are in a windowless room^a^	
4. Locating your car in a very large parking lot or parking garage^a^	
5. Trying a new route that you think will be a shortcut, without the benefit of the map^a^	
6. Finding your way back to a familiar area after realizing you have made a wrong turn and become lost while driving^a^	
7. Finding your way around in an unfamiliar mall^a^	
8. Finding your way to an appointment in an unfamiliar city or town^a^	
9. Constructing a tent at the beach	
10. Following origami paper folding instructions	
11. Building a Lego Architecture® Empire State building using the instructions	
12. Playing Tetris®	
13. Folding flattened cardboard into a gift box by following the folds/creases	
14. Untangling severely tangled headphone cords	
15. Building a 6-drawer dresser from IKEA by following the diagram	
16. Solving a 1000-piece puzzle	
17. Constructing a model house using Legos using only an image of the end product	
18. Packing a trunk with limited space and a lot of objects	
19. Packing a carry-on suitcase with many belongings	
20. Moving all of your furniture from a larger space into a smaller space	
21. Hanging up several pictures, frames, or decals on a wall	

*Note.*^a^ indicates the original items produced by Lawton (1994)

#### State-Trait Anxiety Inventory

The trait subscale (*STAI-T)* of the *State-Trait Anxiety Inventory (STAI), Form Y* (Spielberger, 1983) was used to measure participants’ general anxiety. This questionnaire consisted of 20 statements: nine statements about an anxiety absent feeling (e.g., “I feel pleasant”) and 11 statements about how much anxiety one feels (e.g., “I feel like a failure”) on any given day (i.e., anxiety present statements). Participants indicated their agreement with these statements on a 4-point scale where 1 = Not at all, 2 = Somewhat, 3 = Moderately So, and 4 = Very Much So. The eight statements regarding an anxiety absent feeling (e.g., “I feel pleasant) were reverse coded to match the scale of the anxiety present statements (e.g., “I feel like a failure) allowing us to calculate a composite score for trait anxiety. Scores on all statements were aggregated to calculate a trait anxiety score (range: 20 - 80). We refer to this variable as trait anxiety in all analyses and use it as a control for general anxiety. The STAI-T has a high internal reliability (*α* = 0.86) and correlates with other measures of anxiety such as *Scheier’s Anxiety Scale Questionnaire* (*r* = 0.85; Julian, 2011).

## Results

A final sample of 517 participants (357 females, 160 males) was used for all analyses. The MRT (mental rotation) scores, M-SAS (spatial anxiety) scores, and trait anxiety scores reported were standardized. Descriptive statistics by participant sex are demonstrated in Table 2.

**Table 2:** Means and standard deviations of the variables by gender

Variable	Females		Males		All			
	*M*(*SD*)	*N*	*M*(*SD*)	*n*	*M*(*SD*)	*n*	Minimum	Maximum
Mental rotation test score	10.45 (6.47)	357	14.24 (7.35)	160	11.62 (6.94)	517	0	24
Spatial anxiety	44.07 (10.10)	357	40.21 (10.24)	160	42.95 (10.40)	517	21	79
Navigation anxiety	18.10 (4.52)	357	15.68 (4.70)	160	17.35 (4.71)	517	8	32
Mental rotation anxiety	15.91 (5.15)	357	15.24 (5.23)	160	15.70 (5.18)	517	8	32
Visualization anxiety	6.45 (2.25)	357	5.78 (2.09)	160	6.25 (2.22)	517	3	12
Trait anxiety	42.50 (10.88)	357	38.30 (9.82)	160	41.22 (10.78)	517	20	73

*Note.* Gender was coded as 0 for female and 1 for male. Navigation anxiety, mental rotation anxiety, and visualization anxiety are all sub-factors that make up the Spatial anxiety measure

### Sex differences in mental rotation, spatial anxiety, and trait anxiety

A multivariate analysis of variance (MANOVA) was conducted to assess sex differences in mental rotation, spatial anxiety, and trait anxiety, while accounting for the correlation between the dependent variables (Tabachnick, Fidell, & Ullman, 2007). Prior to conducting the MANOVA, a series of Pearson correlations were conducted between all the dependent variables to test the assumption that all dependent variables would be moderately correlated (Meyers, Gamst, & Guarino, 2006). Table 3 demonstrates a significant correlation between mental rotation and spatial anxiety, as well as spatial anxiety and trait anxiety. Interestingly, trait anxiety did not significantly correlate with mental rotation.

**Table 3:** Pearson correlation with mental rotations score, spatial anxiety, navigation anxiety, mental rotation anxiety, and trait anxiety

	1	2	3	4	5	6
1. Mental rotation test score	*48.22*					
2. Spatial anxiety	−0.20***	*108.08*				
3. Navigation anxiety	−0.13**	0.78***	*22.17*			
4. Mental rotation anxiety	−0.22***	0.83***	0.38***	*53.81*		
5. Visualization anxiety	−0.10*	0.70***	0.45***	0.45***	*4.93*	
6. Trait anxiety	−0.04	0.26***	0.25***	0.16***	0.20***	*116.19*
*M*	11.62	42.95	17.35	25.60	6.25	41.22
*SD*	6.94	10.40	4.71	7.34	2.22	10.78

*Note.* *p ≤ 0.05, **p ≤ 0.01, ***p ≤ 0.001; Italicized numbers on the diagonal are variances, those below the diagonal are correlations

To test the assumption of covariance of matrices, Box’s *M* value = 75.780 was found to be significant (*p* < 0.001), indicating unequal covariance matrices within the variables. The assumption of homogeneity of variance was checked using Levene’s F Test, which was satisfactory for spatial anxiety, trait anxiety, and other demographic data, including participant age, participant grade point average, and survey duration. However, mental rotation and other demographics, including years spent in higher education and the number of years of mathematics and statistics courses taken, were significant (*p* < 0.001) indicating that these variables have variances that are not homogenous. Additionally, the Shapiro-Wilks test indicated that the mental rotation was not normally distributed.

As recommended by Moder (2010), a Welch’s analysis of variance was conducted on the variables that violated the assumption of equal variances. Welch’s test revealed significant sex differences in mental rotation and years participants spent in higher education; however, no significant sex differences were observed in the amount of mathematics and statistics courses that participants reported having taken. Considering the violation of homogeneity of variance and normality with mental rotation*,* Pillai’s trace was used to assess the significance of the MANOVA as it is the most robust test when assumptions are not met.

The *MANOVA* was conducted using participant sex as a fixed factor and the dependent variables were years in higher education, participant age, years spent in mathematics and statistics classes, participant grade point average, survey duration, mental rotation, spatial anxiety, and trait anxiety. This analysis was chosen to reduce the chance of committing a type 1 error by accounting for the correlation between the dependent factors. Pillai’s trace test was chosen as the data did not meet all the assumptions for a MANOVA. A significant difference was observed between male and female scores (Pillai’s trace = 0.158, *F* (8, 501) = 9.14, *p* < .001,

${\eta}_p^2$= 0.127) for years in higher education, participant age, years spent in mathematics and statistics classes, participant grade point average, survey duration, mental rotation, spatial anxiety, and trait anxiety.

Univariate tests demonstrate a significant sex difference in mental rotation (*F* (1,508) = 34.35, *p* < 0.001, ${\eta}_p^2$ = 0.06), with male participants (*M* = 14.24, *SD* = 7.35) on average scoring higher than female participants (*M* = 10.45, *SD* = 6.47) . Significant sex differences also were observed in spatial anxiety (*F* (1, 508) = 15.89, *p* < .001, ${\eta}_p^2$ = 0.03), with female participants scoring higher (*M* = 44.07, *SD* = 10.10) than male participants (*M* = 40.21, *SD* = 10.24). Trait anxiety was also significantly different by participant sex (*F* (1, 508) = 17.25, p < .001, ${\eta}_p^2$ = 0.03), with female participants (*M* = 42.50, *SD* = 10.88) reporting higher trait anxiety than male participants (*M* = 38.30, *SD* = 9.82). The number of years spent in higher education also demonstrated sex differences (*F* (1, 508) = 4.96, *p* < .05, ${\eta}_p^2$ = 0.01), with female participants (*M* = 3.05, *SD* = 1.47) reporting having spent more years in higher education than male participants (*M* = 2.72, *SD* = 1.70).

Finally, a non-parametric *Kruskal-Wallis test*, a test that does not require the assumption of equal variances to be met, was conducted on the mean ranks of the dependent variables which confirmed the findings from the univariate tests.

### Aim 1: Exploring the factor structure of M-SAS using exploratory factor analysis

**Fig. 1:**
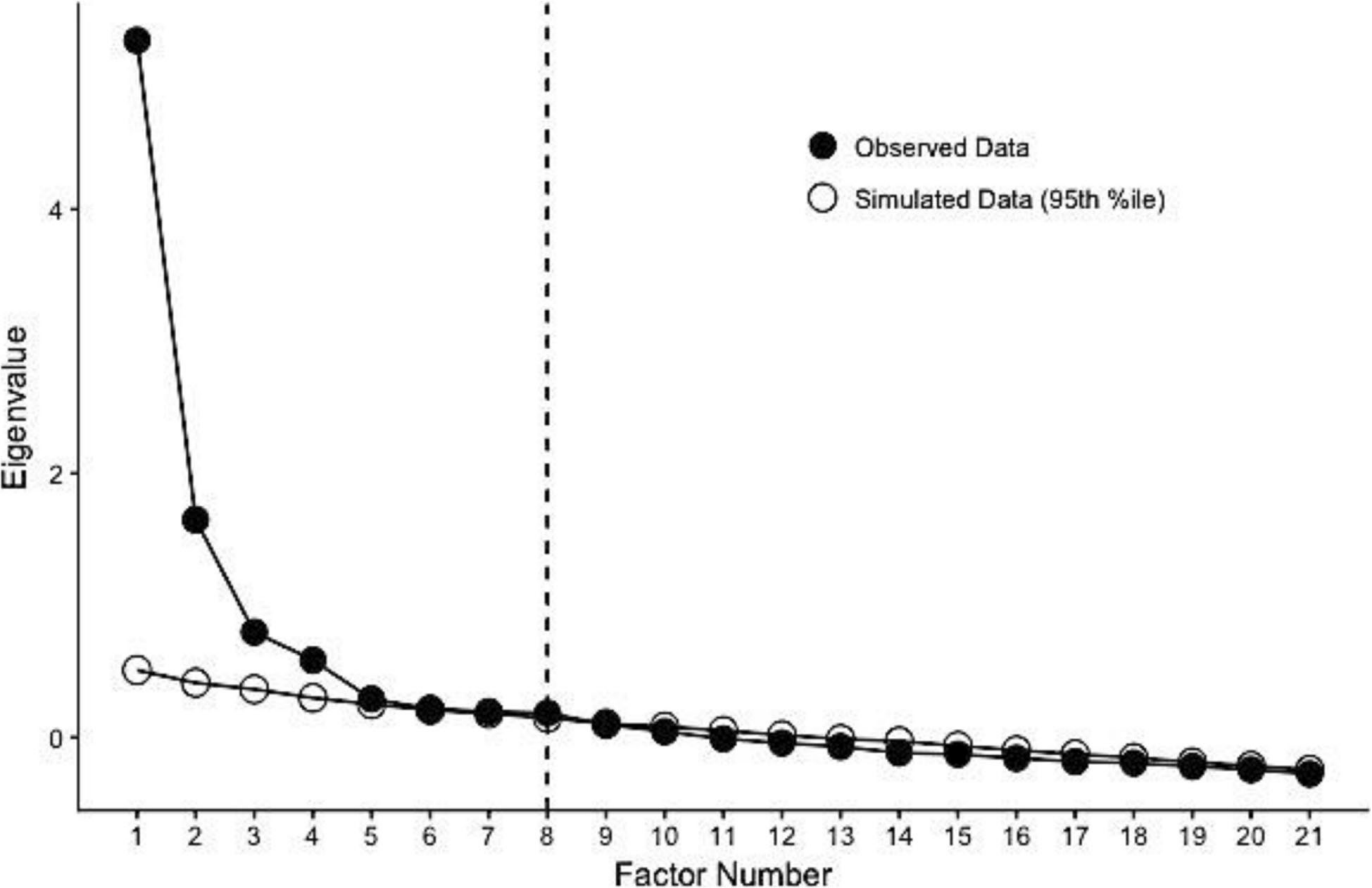
Parallel analysis

Using Mplus (Version 8, Muthén & Muthén, 2018) statistical software, all 21 categorical items from the M-SAS were entered into an exploratory factor analysis. Maximum likelihood extraction was used as all items were expected to be related, and an oblique rotation was specified. Three factors with eigenvalues greater than 1.00 were extracted. A parallel analysis, shown in Fig. 1, was used to ensure that the eigenvalues exceeded values that would be retained from a random dataset of a comparable magnitude (DeVellis, 2016). This analysis yielded a correct estimation of four separate factors that are statistically significant. However, two items did not load onto a specific factor beyond the cutoff value of 0.5 as recommended by Costello and Osborne (2005).

Model fit was assessed using the following criteria: Tucker Lewis index (TLI) > 0.90 (Tucker & Lewis, 1973), comparative fit index (CFI) > 0.90 acceptable (Bentler, 1990), and root mean square error of approximation (RMSEA) < 0.08 (Brown & Cudeck, 1993). The results of all iterations of exploratory factor analysis are shown in Table 4. The three factors were most adequate with a CFI = 0.94, TLI = 0.92, and a RMSEA = 0.07 with CI [0.06, 0.07] and probability RMSEA = 0.000. The eight items from the original Spatial Anxiety Scale (SAS) developed by Lawton (1994) loaded onto Factor 1, which explained 41% of the variance. Factor 2 consisted of eight items, two of which cross loaded onto Factor 3 (items 15 and 17). Factor 2 explained 34% of the variance, and Factor 3 consisted of three other additional items, which taken altogether explained 25% of the variance.

**Table 4:** Items retained in the Modified Spatial Anxiety Scale (M-SAS)

		Factor		
		1	2	3
1.	Leaving a store that you have been to for the first time and deciding which way to turn to get to a destination ^a^	0.72		
2.	Finding your way out of a complex arrangement of offices that you have visited for the first time ^a^	0.79		
3.	Pointing in the direction of a place outside that someone wants to get to and has asked you for directions, when you are in a windowless room ^a^	0.57		
4.	Locating your car in a very large parking lot or parking garage. ^a^	0.47		
5.	Trying a new route that you think will be a shortcut, without the benefit of the map ^a^	0.65		
6.	Finding your way back to a familiar area after realizing you have made a wrong turn and become lost while driving ^a^	0.58		
7.	Finding your way around in an unfamiliar mall ^a^	0.51		
8.	Finding your way to an appointment in an unfamiliar city or town ^a^	0.59		
9.	Constructing a tent at the beach		0.52	
10.	Following origami paper folding instructions		0.82	
11.	Building a Lego Architecture® Empire State building using the instructions		0.87	
12.	Playing Tetris®		0.58	
13.	Folding flattened cardboard into a gift box by following the folds/creases		0.44	
16.	Solving a 1000-piece puzzle		0.57	
17.	Constructing a model house using Legos using only an image of the end product		0.54	
18.	Packing a trunk with limited space and a lot of objects^b^			0.73
19.	Packing a carry-on suitcase with many belongings^b^			0.87
20.	Moving all of your furniture from a larger space into a smaller space^b^			0.69

*Note*. ^a^ Indicates the original items produced by Lawton (1994)

^b^ Visualization factor explored in later analysis should be interpreted with caution, as we had fewer than five items loading onto this factor

Items 14 and 21 were removed as they did not load onto any factors above the cut–off of 0.5. As recommended by Tabachnick and Fidell (2001), the two items that cross loaded onto two factors, items 15 and 17, were kept but reassessed in a follow-up exploratory factor analysis that was conducted with the remaining 19-items (all but items 14 and 21). The remaining 19 categorical items were entered into an EFA maximum likelihood extraction, oblique-specified. Four factors with eigenvalues greater than 1.00 were extracted; however, when the model with three factors was compared to the model with four factors, the three-factor model clearly held the cleanest factor structure. All of the EFA models conducted were compared and can be found in Supplementary Table 1. The model fit was adequate with a comparative fit index (CFI) = 0.95, and a root mean square of error approximation (RMSEA) = 0.07, CI [0.06, 0.08], Probability RMSEA = 0.000. Item 15 still cross loaded in the second EFA model, so this item was removed to determine if the remaining items loaded in a cleaner manner. A final EFA was conducted yielding the cleanest 18-item, three-factor model with a χ^2^(102) = 361.574, CFI = .94, TLI = .92, and RMSEA = .07, CI [0.06, 0.08], probability RMSEA = 0.000.

The EFA yielded a final 18-item measure of spatial anxiety, with all items and factor loadings shown in Table 4. Factor 1 included eight items measuring navigation anxiety; Factor 2, the following seven items measuring mental rotation anxiety; and Factor 3, the last three items measuring visualization anxiety (tasks requiring spatial thinking without the assistance of a concrete example for reference). However, Costello and Osborne (2005) warn that more than five strong loading factors are preferred to specify a single factor; thus, we cannot be certain that the items loading onto Factor 3, visualization anxiety, actually represents a latent type of spatial anxiety. Further analyses with visualization anxiety should be interpreted with caution.

Thus, we extracted factors representing navigation anxiety and mental rotation anxiety, much like others have previously found (Lyons et al., 2018; Malanchini et al., 2017), in addition to a third factor, visualization anxiety. Items on navigation anxiety characterized tasks that require the visualization of a large-scale external environment from different points of reference. In contrast, mental rotation anxiety items were characterized by tasks requiring the mental transformation, visualization, or configurations of 2D and 3D objects, typically when the end result of the object being manipulated is well-known or present. In contrast, the items loading onto the third factor, visualization anxiety, required the visualization of the spatial configuration of objects without the assistance of a concrete reference. The internal consistency of each factor was assessed using Cronbach’s Alpha for Factor 1 ∝ = 0.80, Factor 2 ∝ = 0.80, and Factor 3 ∝ = 0.74.

### Aim 2: Examine whether sex differences in MRT performance are mediated by individual differences in spatial anxiety

We first examined whether sex differences on the MRT could potentially be explained by our modified spatial anxiety measure, one that collapsed across the remaining 18-items, while controlling for trait anxiety. Before conducting our mediation, a multiple regression analysis revealed the potential mediation effect of all the dependent variables on the relation between participant sex and mental rotation (β = 0.228, t = 5.53, *p* < 0.001, *R*^*2*^ = 0.05). Each mediator was regressed on participant sex, and each subsequent dependent variable was regressed on the suggested mediator. Results showed that participant sex was significantly correlated to spatial anxiety (β = − 0.162, *t* = − 3.86, *p* < .001, *R*^*2*^ = 0.02). Similarly, spatial anxiety was significantly related to mental rotation (β = − 0.207, *t* = −5.00, *p* < 0.001, *R*^*2*^ = 0.04).

We also examined trait anxiety as a potential moderator. Participant sex was correlated to trait anxiety (β = − 0.184, *t* = − 4.42, *p* < 0.001, *R*^*2*^ = 0.03), and trait anxiety was significantly related to spatial anxiety (β = 0.28, *t* = 6.90, *p* < 0.001, *R*^*2*^ = 0.08). However, trait anxiety did not explain mental rotation (β = − 0.064, *t* = −1.52, *p* > 0.05, *R*^*2*^ = .0004). Since trait anxiety is significantly related to spatial anxiety but not to mental rotation, we included it as a potential moderator. Pearson correlations (Table 3) support the prerequisites for the mediation analysis among participant sex, mental rotation, spatial anxiety, and trait anxiety.

A simple mediation model was implemented on the PROCESS Macro (Version 2.16.3; Hayes, 2009, 2013) for IBM SPSS (version 20). All models mentioned control for participant age, years spent in higher education, amount of math and statistics courses taken, and participant grade point average. The significance of each effect was assessed using a bias-corrected bootstrapped 95% confidence interval based on 5000 bootstrapped samples, as recommended by Preacher and Hayes (2008). If a significant effect exists in a single path in the mediation analysis, then the confidence interval of the effect should not include the value of zero. Accordingly, the effects reported from the simple mediation do not include a zero within the confidence interval.

**Fig. 2:**
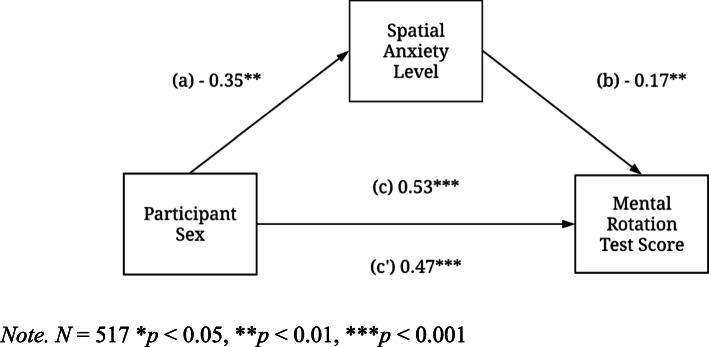
Direct and indirect effect of participant sex on mental rotation test scores

The effect of participant sex on mental rotation was significant and reported as a standardized regression coefficient (*c* = 0.53, *t* (515) = 5.79, *p* < .001, 95% CI [0.35, 0.71]). Participant sex was entered as a dichotomous variable, for ease of regression coefficient interpretation, where female participants = 0 and male participants = 1. Thus, the positive coefficient of the direct effect of participant sex on mental rotation test scores indicates that male participants, on average, scored 0.53 standard deviations higher mental rotation scores than female participants. The strength of this effect (path *c*) is shown in Fig. 2.

The potential mediation path was assessed by examining the joint significant paths as recommended by MacKinnon, Lockwood, Hoffman, West, and Sheets (2002). The association between participant sex and spatial anxiety (path *a* = − 0.35, *t* (515) = − 4.71, *p* < .001, 95% CI [− 0.53, − 0.16]) was negatively and statistically significant, indicating a negative relation between participant sex and spatial anxiety in which female participants (dummy coded as 0) had 0.35 higher levels of spatial anxiety. The influence of spatial anxiety on mental rotation (path *b* = − 0.17, *t* (515) = − 3.95, *p* < .001, 95% CI = [− 0.25, − 0.08]) is also negative and significant. Figure 2 demonstrates that when holding participant sex constant, each unit increase in spatial anxiety was associated with a 0.17 mental rotation unit drop. Put simply, participants with higher levels of spatial anxiety had lower mental rotation test scores.

The estimated indirect effect of participant sex on mental rotation through level of spatial anxiety is *b = (− 0.35) (− 0.17) = 0.06, meaning that male participants scored 0.06 points higher than female participants as a result of the negative effect of participant sex on level of spatial anxiety. For female participants, this increase (0 + 0.06 = 0.06) in mental rotation scores is less than that of male participants (1 + 0.06 = 1.06). The estimated effect of participant sex on mental rotation (path *c’* = .47, t (515) = 5.16, *p* = 0.00, 95% CI [0.29, 0.65]) is still significant but less than with path *c,* suggesting mediation. This finding indicated that, in a hypothetical group of male and female participants with equal levels of spatial anxiety, a male advantage of 0.47 units would still be present in the MRT.

### Moderated mediation: what role does trait anxiety play?

As noted above, trait anxiety was not significantly related to mental rotation, yet it was correlated to participant sex and spatial anxiety. To determine if the relation between participant sex, spatial anxiety, and mental rotation was contingent on the level of trait anxiety, we conducted a moderated mediation using model 8 from the PROCESS Macro (Hayes, 2012).

**Fig. 3:**
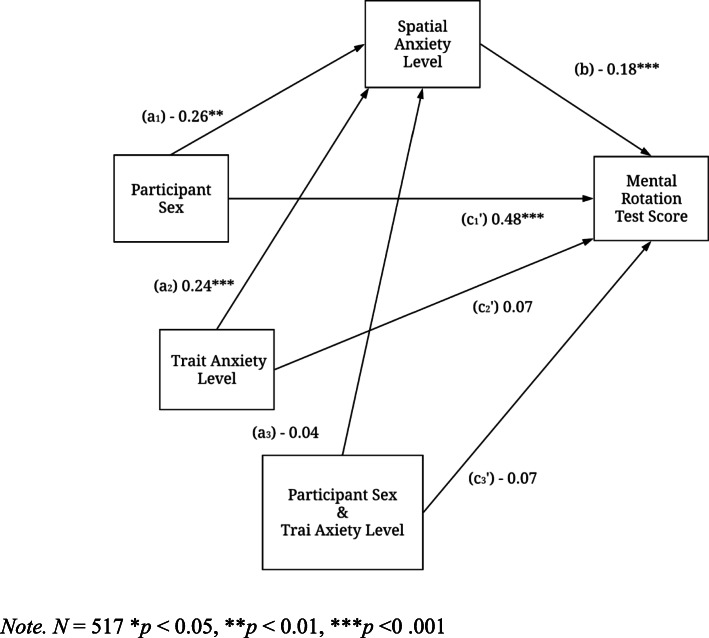
Statistical model of moderated mediation

To determine whether trait anxiety influenced the relation between participant sex, spatial anxiety, and mental rotation, we entered trait anxiety as a moderator. Figure 3 shows the statistical analyses including the association between participant sex, trait anxiety*,* spatial anxiety, and mental rotation. As shown in Fig. 3, the relation between participant sex and spatial anxiety (path *a*_*1*_ = − 0.26, *t* (513) = − 2.79, *p* = .005, 95% CI [− 0.45, − 0.08]) was reduced when trait anxiety was entered. This suggested that trait anxiety influenced the negative relation between participant sex and spatial anxiety*.* Furthermore, trait anxiety had a significant positive association with spatial anxiety (path *a*_*2*_ = 0.24, *t* (513) = 4.85, *p* < .001, 95% CI [0.15, 0.34]), with each unit of trait anxiety showing a 0.24 increase in the level of spatial anxiety. When participant sex and trait anxiety were held constant, the effect of spatial anxiety on mental rotation was significant (path *b* = − 0.18, *t* (513) = − 4.12, *p* < .001, 95% CI = [− 0.27, − 0.09]). Similarly, the direct effect of participant sex on mental rotation (path *c’* = .48, *t* (513) = 5.10, *p* < .001, 95% CI [0.29, 0.66])was reduced but still significant.

Trait anxiety did not significantly moderate the mediation of spatial anxiety (path *a*_*3*_ = − 0.04, *t* (512) = 1.36, *p* = 0.17, 95% CI [− 0.03, 0.17]). The interaction between participant sex and trait anxiety (path *c*_*3*_’ = − 0.07, *t* (512) = − 0.68, *p* = 0.50, 95% CI [− 0.26, 0.12]) demonstrates that the relation between participant sex and mental rotation does not depend on the trait anxiety.

### Aim 3: Explore whether mental rotation and/or navigation anxiety mediates the sex difference in performance on the MRT

**Fig. 4:**
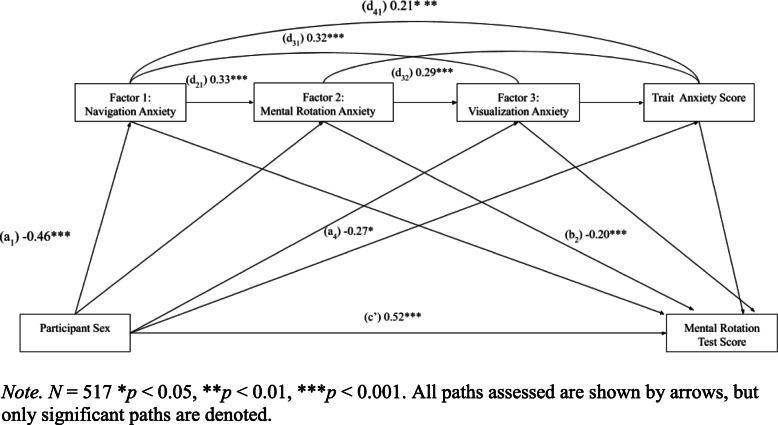
Mediation of the relation between participant sex and mental rotation score including factors identified through exploratory factor analysis and trait anxiety score

Finally, we investigated whether mental rotation anxiety as a specific construct (rather than navigation anxiety) mediated the sex difference in MRT. Model 6 from the PROCESS Macro (Hayes, 2012) was used, which allows the inclusion of multiple mediators to assess mediation of each individual factor identified through our EFA. The model shown in Fig. 4 was conducted while controlling for participant age, number of years in higher education, the number of math and statistics courses taken, and grade point average. The mediated effect accounted for approximately 11% of the variability in mental rotation performance (*R*^*2*^ = 0.11, F (9,500) = 6.64, *p* < 0.001). Unlike ordinary least square regressions, the *R*^*2*^ in the mediation model is used to compare the relative effect sizes of the component paths in the model and isolate the variance in mental rotation performance accounted for by the mediated effect (Fairchild, Mackinnon, Taborga, & Taylor, 2009). Since the mediation model implemented uses bootstrapping with maximum likelihood procedures, and the component paths from covariates are not shown in the diagram, the component paths cannot be squared to get the *R*^*2*^.

The relation between participant sex and mental rotation remained significant even after entering in multiple mediators (path *c’* = 0.52, *t* (502) = 5.44, *p* < 0.001, 95% CI [0.33, 0.71]). However, we identified a significant indirect effects of participant sex on mental rotation through Factor 1: navigation anxiety and factor 2*:* mental rotation anxiety*.* Participant sex was significantly associated with Factor 1: navigation anxiety (path *a*_*1*_ = − 0.46, *t* (503) = 4.98, *p* < .001, 95% CI [− 0.64, − 0.28]). Navigation anxiety then was positively significantly associated to mental rotation anxiety (path *d*_*21*_ = 0.33, *t* (502) = 7.58, *p* < 0.001, 95% CI [0.25, 0.42]), such that individuals with higher navigation anxiety are also more likely to have higher mental rotation anxiety. However, mental rotation anxiety was the only mediator entered that was significantly associated with the Mental Rotations Test scores (*path b*_*2*_ = − 0.20, *t* (500) = − 4.25, *p* < 0.001, 95% CI [− 0.30, − 0.11]). We interpret this as an indirect effect of participant sex on navigation anxiety which predicts mental rotation anxiety which then predicts Mental Rotations Test scores.

Interestingly, Factor 1 or navigation anxiety (path *b*_*1*_ = − 0.02, *t* (500) = − 0.38, *p* = 0.70, 95% CI [− 0.11, 0.08]) and Factor 3 or visualization anxiety (path *b*_3_ = − 0.02, *t* (500) = 0.39, *p* = 0.71, 95% CI [− 0.08, 0.12]) alone did not significantly mediate the relation between participant sex and mental rotation. Similarly, trait anxiety did not mediate the relation between participant sex and mental rotation (path *b*_*4*_ = − 0.04, *t* (500) = 0.81, *p* = 0.42, 95% CI [− 0.05, 0.12]). However, the amount of trait anxiety reported did significantly relate to participant sex shown (path *a*_*4*_ = − 0.27, *t* (500) = − 4.14, *p* < 0.001, 95% CI [− 0.58, − 0.21]) and the amount of Factor 1 or navigation anxiety, by another (path *d*_*41*_ = 0.21, *t* (500) = 4.85, *p* < 0.001, 95% CI [0.12, 0.29]). These results indicate that female participants reported higher levels of trait anxiety and navigation anxiety (anxiety felt when confronted with a task in which one must navigate a 3D environment anxiety); however, the sex differences in both trait anxiety and navigation anxiety did not explain the sex differences in the mental rotation.

## Discussion

The aims of the current study were to (1) develop a Modified Spatial Anxiety Scale (M-SAS) that included items that corresponded to navigation and mental rotation to explore the factor structure of this newly developed scale, (2) to investigate whether reported sex differences in mental rotation are mediated by individual differences in spatial anxiety using this newly developed M-SAS, and (3) to examine whether specific factors identified using the exploratory factor analysis (i.e., navigation anxiety, mental rotation anxiety, visualization anxiety) mediate sex differences in mental rotation.

To determine whether spatial anxiety explained sex differences in performance on the MRT, we modified the Spatial Anxiety Scale (SAS; original developed by Lawton, 1994), conducted an exploratory factor analysis to compare the factor loadings with recent work on the factorial structure of spatial anxiety (Lyons et al., 2018; Malanchini et al., 2017), and analyzed its influence through a series of mediation models. An exploratory analysis on the factor structure of the M-SAS identified three separate factors, which corresponded to: (1) navigation anxiety, (2) mental rotation anxiety; and (3) an abstract form of spatial thinking or what we called visualization anxiety. Our results align with Lyons et al. (2018) in that we were able to find factors related to both spatial navigation and spatial-mental manipulation/rotation. We also, however, found a third factor that did not quite align with prior studies, that we termed visualization anxiety. Lyons and colleagues explored the factorial structure of spatial anxiety with more items that align closely to the 2 × 2 spatial typology (i.e., extrinsic-intrinsic; dynamic-static) proposed by Uttal et al. (2013). Our scale did not include imagery items, such as “such as recalling the color of someone’s tie” which may explain why the third factor we identified does not align with the imagery factor from Lyons et al. (2018). Instead, our third factor consisted of items regarding the visualization of abstract objects, such as “packing a carry-on suitcase with many belongings”; which may incorporate both of the intrinsic-static (recognizing the spatial configuration of an static object) and the extrinsic-static (comparing the location of static objects in an environment) features of visual stimuli provided by the framework of Uttal et al. (2013). We do caution the reader in interpreting our results on the third factor, visualization anxiety, as we had only three items loading onto this factor, and some have warned that to specify a latent factor one needs more than five strong items loading onto that factor (Costello & Osborne, 2005).

In agreement with previous literature, men outperformed women on the mental rotation test (Linn & Petersen, 1985; Maccoby & Jacklin, 1974; Nazareth et al., 2013; Uttal et al., 2013; Voyer et al., 1995). Furthermore, female participants scored significantly higher than male participants in both spatial anxiety (Lawton & Kallai, 2002) and trait anxiety (Bander & Betz, 1981). The correlation between mental rotation performance and the M-SAS (−.20) is slightly stronger than the correlation between mental rotation performance and the original SAS of −.14 (Lawton, 1994). Additionally, the correlation between spatial anxiety and trait anxiety (.28), as measured by the trait anxiety subset, is similar to the correlation between trait anxiety and Lawton and Kallai’s way-finding anxiety scale (.29) for the American sample (2002). Our M-SAS may be more strongly correlated with mental rotation performance due to the addition of anxiety items that pertain to situations about mentally rotating and manipulating tangible objects.

Spatial anxiety, when considered as one comprehensive construct, was found to partially mediate the relation between participant sex and mental rotation performance, while controlling for participant age, grade point average, and years spent in higher education. Trait anxiety was entered into the mediation model as a moderator to determine if the influence of spatial anxiety on mental rotation test scores was due to the level of trait anxiety. The results suggest spatial anxiety explains sex differences in mental rotation test scores beyond the influence of trait anxiety. Importantly, trait anxiety, our measure of general anxiety, did not explain the sex differences in mental rotation test scores. Furthermore, spatial anxiety was not necessarily dependent on a person’s level of trait anxiety, providing evidence that there may be individual differences in domain-specific anxieties, such as spatial anxiety, which are not fully explained by generalized anxiety. The literature has supported the prevalence of underlying skills that make up spatial ability; therefore, domain-specific anxieties possibly exist that each explain individual differences in domain-specific skill (Lauer et al., 2018).

Finally, we conducted a multiple mediator analysis to explore whether one particular type of spatial anxiety was explaining sex differences in mental rotation. We considered navigation anxiety, mental rotation anxiety, visualization anxiety, and trait anxiety as mediators of the relation between participant sex and MRT scores. This analysis revealed that the anxiety felt towards tasks requiring navigation and mental rotation performance mediated sex differences on the MRT. Navigation anxiety alone did not explain the relation between participant sex and mental rotation ability, perhaps because navigation anxiety is more burdensome on tasks that require navigation rather than tasks requiring mental rotation. However, the anxiety felt towards navigation was strongly related to the anxiety felt towards mental rotation. Navigation anxiety did elicit the strongest relation with participant sex and trait anxiety, suggesting that on average females have higher levels of navigation and trait anxiety. Lawton (1994) reported that people who use the orientation way-finding strategy are more likely to become confused about their sense of position in their environment which may explain the negative correlation between spatial anxiety and orientation way-finding strategy. This provides an explanation for the mechanism through which navigation anxiety may impact performance on way-finding; however, the mechanism underlying the association of navigation anxiety and mental rotation anxiety and mental rotation performance is not well understood. The relation between navigation and mental rotation anxiety may be influenced by strategy choice, performance in, and perception of ability in navigation and mental rotation tasks.

A separate possibility is that participants with navigation anxiety may avoid tasks requiring spatial skills like navigation and mental rotation. This has been documented in participants with high math anxiety, where the avoidance of math may reduce a participants’ opportunity to improve their math skills (Hembree, 1990). If a person feels anxious when engaged with spatial activities, they, reasonably, may avoid such activities; this would limit the development of that individual’s spatial skills. Thus, we find that spatial anxiety mediates the relation between participant sex and mental rotation test scores through two potential mechanisms, where (1) anxious ruminations may interfere with mental rotation performance and/or (2) a participant’s own knowledge of their anxiety may evoke their distancing from potential opportunities to hone their mental rotation skills.

### Limitations

An important limitation of this study is the absence of an experimental design that can discern the causal impact of spatial anxiety on performance on the MRT. Our correlational findings, however, highlight the potential of exploring how domain-specific spatial anxieties such as mental rotation anxiety hinder performance in the MRT. Furthermore, we included the trait anxiety subscale (STAI-T; Spielberger, 1983) as a control measure of how anxious people feel in general, but we did not include the state anxiety measure which could help parse out how much anxiety is felt in general*,* in contrast to at the present moment. Similarly, the test anxiety inventory could be included in future work to determine which individuals are specifically nervous about being in a testing situation that is not captured through trait anxiety. The inclusion of a test anxiety measure would also serve to assess the construct validity of the M-SAS. Finally, our visualization anxiety factor had only included three items, so interpreting results regarding this particular domain-specific spatial anxiety is difficult.

### Future directions

To better understand the mechanisms underlying the correlation between domain-specific anxiety and mental rotation performance, future work should include different measures of mental rotation and strategy use. If domain-specific spatial anxieties do differentially impact performance on domain-specific measures, we would expect that mental rotation anxiety would be more negatively associated with measures of mental rotation ability than measures of navigation or visualization ability. However, this has yet to be tested. Sex differences in the strategies used to complete a spatial task are correlated with performance in navigation (Lawton, 1994) and mental rotation performance (Nazareth et al., 2019). Future work can determine how domain-specific anxieties relate to strategy choice and whether changing the strategy used to solve a spatial problem influences performance or levels of domain-specific spatial anxiety. Such work would clarify the pathways through which intervention could reduce sex differences in navigation and mental rotation performance, which is important, as short-term interventions have successfully increased undergraduate students mental rotation skills, yet they have not bridged the sex differences in mental rotation performance (Miller & Halpern, 2013; Sorby et al., 2013).

Moreover, Attentional Control Theory (ACT; Derakshan & Eysenck, 1998; Eysenck & Derakshan, 2011) poses that the link between anxiety and performance can explained by the modulation of anxiety on attentional processes such as working memory. Beilock (2008) found that people with high working memory capacity are most affected by stressful-situation induced worries on math performance. Considering the link between math performance and mental rotation ability, future work can manipulate working memory to determine if the relation between spatial anxiety and mental rotation performance is due to working memory capacity. Experimental designs would be optimal to distinguish the extent to which mental rotation anxiety, navigation anxiety, strategy selection, and working memory separately hinder mental rotation performance or other spatial thinking tasks. By including various measures of general anxiety and domain-specific anxieties, like spatial anxiety and math anxiety, we can begin to unpack the relation between anxiety and individual differences in spatial ability. Finally, we think our data suggest that domain-specific anxiety, that is, spatial anxiety, should be treated as a potential variable to change in an intervention study.

## Conclusion

Spatial ability serves as a gatekeeper for success in entry-level STEM courses; however, consistent sex differences have been found with males outperforming females on measures of one specific type of spatial ability, mental rotation ability. We demonstrate that spatial anxiety mediates the relation between participant sex and mental rotation performance. Furthermore, we explore the factorial structure of spatial anxiety and find separate factors comprise spatial anxiety, mental rotation anxiety, navigation anxiety, and possibly visualization anxiety. Finally, we show that when these specific spatial anxieties are considered as mediators of the relation between participant sex and mental rotation performance, only mental rotation anxiety mediates the sex difference in mental rotation performance.

## Supplementary information

**Additional file 1.** Fit indices for each exploratory factor analysis model tested.

## Data Availability

Data and materials are available upon request from the first author.

## Abbreviations

STEM: Science, Technology, Engineering, Mathematics
MRT: Mental Rotations Test
SAT-M: Scholastic Aptitude Test for Math
2D: Two-dimensional
3D: Three-dimensional
SAS: Spatial Anxiety Scale
M-SAS: Modified Spatial Anxiety Scale
GAD: Generalized Anxiety Disorder Scale
STAI-Trait: State-Trait Anxiety Inventory Trait Scale
MANOVA: Multivariate analysis of variance
