# Supplementary material for: Spatial anxiety mediates the sex difference in adult mental rotation test performance
Source: Cogn Res Princ Implic. 2020 Jul 25;5:31. doi: 10.1186/s41235-020-00231-8 (PMC7382671; doi:10.1186/s41235-020-00231-8)
Supplement: Supplementary file 1 — Additional file 1. Fit indices for each exploratory factor analysis model tested. [file 41235_2020_231_MOESM1_ESM.docx]

**Supplementary Materials**

| **Supplementary Table 1**  *Fit Indices for Each Exploratory Factor Analysis Model Tested* | | | | | | |
| --- | --- | --- | --- | --- | --- | --- |
| Model Step | χ^2^ | df | p | TLI | CFI | RMSEA |
| Exploratory Factor Analysis (n = 517) | | | | | | |
| 1. 21-items | 490.124 | 150 | <.001 | 0.92 | 0.94 | 0.066 |
| 1. Item 14 and 21 removed | 394.470 | 117 | <.001 | 0.92 | 0.95 | 0.068 |
| 1. Item 15 removed | 361.574 | 102 | <.001 | 0.91 | 0.94 | 0.070 |
|  | | | | | | |
